# Autoimmune [auto-inflammatory] syndrome induced by adjuvants (ASIA): Case report after inguinal hernia repair with mesh

**DOI:** 10.1016/j.ijscr.2021.106060

**Published:** 2021-06-11

**Authors:** Eduardo Rullo Maranhão Dias, Luca Giovanni Antonio Pivetta, João Paulo Venancio de Carvalho, Marcelo Lopes Furtado, Pedro Henrique de Freitas Amaral, Sergio Roll

**Affiliations:** Hernia Center, Gastrointestinal Surgery Service, Oswaldo Cruz German Hospital, São Paulo, SP, Brazil

**Keywords:** Autoimmune [auto-inflammatory] syndrome induced by adjuvants (ASIA), Polypropylene mesh, Inguinal hernioplasty

## Abstract

**Introduction:**

There has been a great advance in the treatment of inguinal hernias with a significant reduction in recurrences with the use of polypropylene mesh. Local complications such as infections, rejection, and chronic pain are widely studied and reported in the literature.

The Autoimmune [Auto-inflammatory] Syndrome Induced by Adjuvants (ASIA) is little known and can be triggered by using polypropylene mesh.

**Presentation of the case:**

33-year-old female patient, married, and an administrative manager. History of smoking, previous breast surgery with silicone prosthesis, appendectomy. One year and four months ago, she underwent bilateral inguinal hernioplasty by laparoscopy. Shortly after the inguinal hernia surgery, systemic, urinary symptoms, and chronic local pain appeared. She reported low back pain, fatigue, memory loss, and mood swings associated with limiting pelvic pain, dysuria, and dyspareunia.

We performed a robotic surgical procedure to remove the meshes bilaterally. Three days after surgery, the patient was discharged with adequate pain control, without the need for opioids. During outpatient follow-up, there was a significant improvement in symptoms, both local and systemic.

**Discussion:**

Local complications with the use of polypropylene mesh to repair inguinal hernias are well described in the literature, highlighting chronic postoperative pain that can affect 10–20% of patients.

Recently, polypropylene prostheses have been found to act as adjuvants and may be the trigger for an exacerbated immune response adaptive to an autoantigen. Thus, being capable of causing an autoimmune disease variant of the Autoimmune [Auto-inflammatory] Syndrome Induced by Adjuvants (ASIA), described by Shoenfeld and Agmon-Levin in 2011.

**Conclusion:**

In addition to local complications, systemic symptoms related to the use of polypropylene mesh can also occur. In the Autoimmune [Auto-inflammatory] Syndrome Induced by Adjuvants (ASIA), systemic symptoms, for being nonspecific, make diagnosis difficult and are often not attributed to the use of mesh.

## Highlights

• A case of mesh related ASIA syndrome
• An autoimmune syndrome related to the use of prosthetics
• Mesh removal must be considered, to achieve control of the symptoms.

## 1 Introduction

The polypropylene mesh allowed great advances in the treatment of hernias of the abdominal wall, significantly reducing recurrences. Despite this important benefit, complications such as infections, rejection, and chronic pain can occur and are widely studied looking for the development of techniques and prostheses that allow better results.

Over the last decade, it was found that the use of prostheses may be the trigger for immunological effects in the organism of the genetically predisposed patient [1]. Symptoms associated with systemic use of these prostheses have been described in the Autoimmune [Auto-inflammatory] Syndrome Induced by Adjuvants (ASIA) – Shoenfeld's syndrome [2]. This syndrome was initially described in the use of other adjuvants, such as the silicone prosthesis. Besides that, there are few reported cases related to the use of polypropylene mesh [3].

The challenge of recognizing this condition is to value symptoms that are often nonspecific, such as myalgia, arthralgia, and fatigue; and to relate the use of prostheses as a triggering factor for these symptoms.

We report herein a case of ASIA after bilateral inguinal hernia repair using a polypropylene mesh. This case report has been reported in line with de SCARE criteria [4], and approved by the ethics committee of Oswaldo Cruz German Hospital, São Paulo, SP, Brazil (register: 45956621.9.0000.0070).

## 2 Case report

The present case report refers to a 33-year-old female patient, married, and an administrative manager. History of smoking, previous breast surgery with silicone prosthesis, appendectomy, no other relevant personal or family history. One year and four months ago, she underwent bilateral inguinal hernioplasty by laparoscopy. Shortly after the inguinal hernia surgery, systemic, urinary symptoms, and chronic local pain appeared. She reported low back pain, fatigue, memory loss, and mood swings associated with limiting pelvic pain, dysuria, and dyspareunia. Moreover, she reported changes in bowel habits in the absence of an organic cause, which had been unsuccessfully treated with trimebutine.

She was then referred to urologist, gynecologist, and rheumatologist specialists for evaluation of the diagnostic hypotheses of interstitial (autoimmune) cystitis and fibromyalgia, starting treatment with pregabalin, fluoxetine, and pentosan polysulfate sodium (Elmiron®).

A tomography of her abdomen and pelvis was performed, which revealed a densification of the anterior bilateral and perivesical inguinal adipose tissue, a non-specific finding, but with a probable inflammatory/reactive etiology. Herniorrhaphy mashes were normal. Directed physical examination revealed bilateral inguinal pain, most notably on the right.

**Fig. 1:**
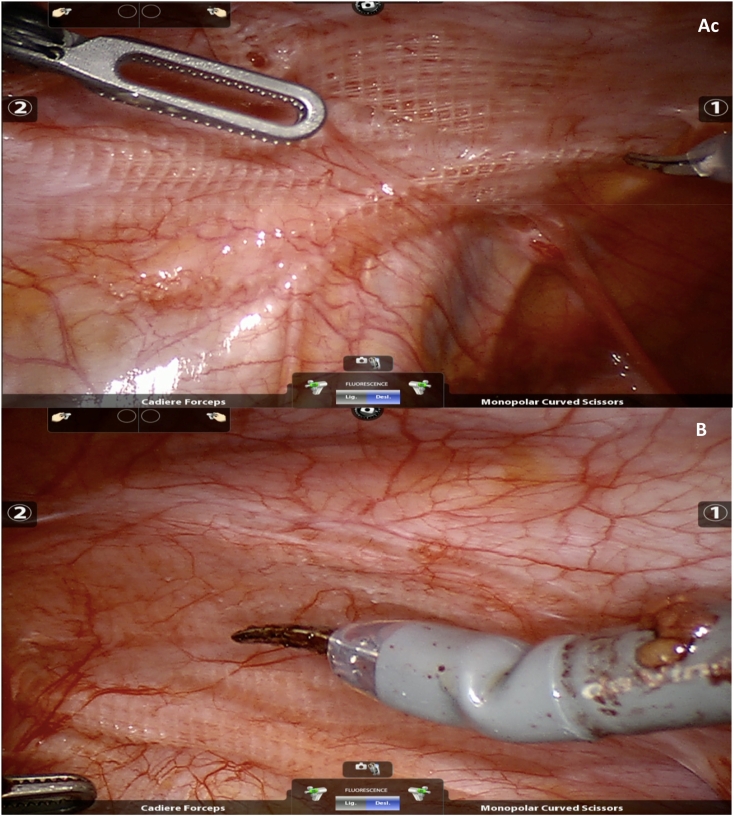
A- view of the left side hernioplasty B- view of the right side hernioplasty.

**Fig. 2:**
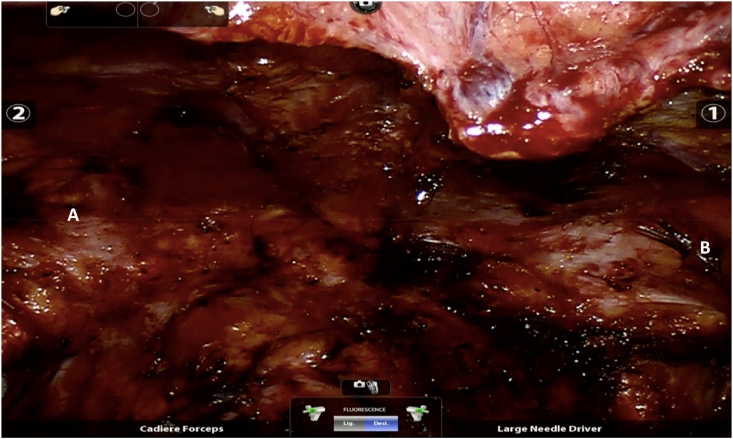
A- Left side after mesh removal B- Right side after mesh removal.

Thus, we performed surgical exploration with robotic approach and removal of the mesh were proposed (Fig. 1). In the procedure, after removing the meshes bilaterally, no significant residual hernial defect was observed (Fig. 2). Three days after surgery, the patient was discharged with adequate pain control, without the need for opioids. No surgical site infection or occurrence was observed.

In the histopathological report, cuts of connective tissue and adipose were observed, showing a slight inflammatory lymphocytic infiltrate and a laminar fibrotic ban. Frequent multinucleated giant cells, characterizing foreign body granuloma to material compatible with surgical mesh were also seen are observed.

During outpatient follow-up- 1 year, there was a significant improvement in symptoms with no inguinal pain, or hernia recurrence, no fatigue or dyspareunia during this period.

## 3 Discussion

Local complications with the use of polypropylene mesh to repair inguinal hernias are well described in the literature, highlighting chronic postoperative pain that can affect 10–20% of patients [5]. One of the justifications is the local inflammatory reaction induced by the polypropylene prosthesis, which can become chronic [6]. In the reported case, there was a chronic limiting local pain in the postoperative period, associated with dyspareunia and dysuria. A local inflammatory process was found in the tomography exam with densification of inguinal and perivesical adipose tissues one year after implanting the polypropylene mesh.

Recently, polypropylene prostheses have been found to act as adjuvants, and may be the trigger for an exacerbated immune response adaptive to an autoantigen [7]. Thus, being capable of causing an autoimmune disease variant of the Autoimmune [Auto-inflammatory] Syndrome Induced by Adjuvants (ASIA), described by Shoenfeld and Agmon-Levin in 2011 [2].

This Syndrome has major and minor diagnostic criteria, which are:

Major criteria

• Exposure to an external stimulus (infection, vaccine, silicone, or adjuvant) before clinical manifestations;
• The appearance of “typical” clinical manifestations:
  o Myalgia, myositis, or muscle weakness;
  o Arthralgia and/or arthritis;
  o Chronic fatigue, non-restorative sleep, or sleep disorders;;
  o Neurological manifestations (especially those associated with demyelination) – Cognitive impairment, memory loss
  o Pyrexia;
  o Xerostomia and/or xerophthalmia;
• Removal of the agent *provocateur* induces significant improvement in symptoms;
• Typical biopsy of the organs involved.

Minor criteria

• The appearance of autoantibodies or antibodies directed at the suspected adjuvant;
• Other clinical manifestations (such as irritable bowel syndrome);
• Specific HLA (HLA-DRB1 or HLA-DQB1);
• Evolution of an autoimmune disease (multiple sclerosis or systemic sclerosis).

Patients are considered to have ASIA when two main criteria or one main and two secondary criteria are present. In the reported case, the patient had typical systemic symptoms of fatigue and myalgia, and the removal of the mesh led to a significant improvement in symptoms. In addition, the biopsy revealed a typical foreign body granuloma reaction. The patient also presented symptoms of irritable bowel syndrome, was diagnosed with interstitial (autoimmune) cystitis and fibromyalgia, all after surgery for inguinal hernia with mesh placement. Thus, the diagnosis of ASIA was confirmed.

In the case reported, local and systemic symptoms appeared shortly after mesh placement. J. W Cohen Tervaert et al., when analyzing 40 cases of ASIA in patients using polypropylene mesh, 32 of whom were female, found 61% starting symptoms in the first year; 25% between one and three years after surgery; and 14% after three years. Like in the case presented, fatigue symptoms were observed in 97.5%; 78% had local pain; and 80% had irritable bowel syndrome. History of allergy was observed in 75% of patients. In the case reported, the patient denied allergies [3].

## 4 Conclusion

In addition to local complications, systemic symptoms related to the use of polypropylene mesh can also occur, and there are few cases reported in the literature. In the Autoimmune [Auto-inflammatory] Syndrome Induced by Adjuvants (ASIA), systemic symptoms, for being nonspecific, make diagnosis difficult and are often not attributed to the use of mesh.

Despite the fact this particular patient has breast implants, and silicone implants is a trigger for development of ASIA, the onset of symptoms, and also the remission after the mesh removal support the hypothesis to in this case ASIA be related to an polypropylene mesh.

Further studies should be carried out to identify predisposed patients, and if silicone implants could predispose to a greater sensibilization after the polypropylene implant and to seek alternatives in the repair of these patients' hernias.

## Funding

This research did not received any specific grant from funding agencies in the public, commercial, or not-for profit sectors.

## Ethics approval

This case report was approved by the ethics committee of Oswaldo Cruz German Hospital, São Paulo, SP, Brazil (register: 45956621.9.0000.0070).

## Informed consent

Informed consent was obtained from the patient.

## International journal of surgery case reports

The following information is required for submission. Please note that failure to respond to these questions/statements will mean your submission will be returned. If you have nothing to declare in any of these categories then this should be stated.

## Registration of research studies

Case reports that are not first-in-man study already approved in Ethics Committee.

## Guarantor

Eduardo R M Dias

## CRediT authorship contribution statement

Eduardo R M Dias, conceptualization, Validation, Writing - Original Draft; Pedro H F Amaral: Methodology, Validation; João P V Carvalho, Formal analysis, Data Curation; Marcelo Lopes Furtado, Formal analysis, Data Curation; Sergio Roll, Writing - Review & Editing, Supervision, Project administration; Luca G A Pivetta, Methodology, Writing - Review & Editing,

## Declaration of competing interest

No conflicts of interest relevant to this article.
